# The Genus *Wallemia—*From Contamination of Food to Health Threat

**DOI:** 10.3390/microorganisms6020046

**Published:** 2018-05-21

**Authors:** Janja Zajc, Nina Gunde-Cimerman

**Affiliations:** 1 National Institute of Biology, Večna pot 111, SI-1000 Ljubljana, Slovenia; janja.zajc@nib.si; 2 Biology Department, Biotechnical Faculty, University of Ljubljana, Jamnikarjeva 101, SI-1000 Ljubljana, Slovenia

**Keywords:** *Wallemia*, food, air, pathogen, xerophile, halophile, mycotoxin, farmer’s lung disease, subcutaneous infection

## Abstract

The fungal genus *Wallemia* of the order *Wallemiales* (Wallemiomycotina, Basidiomycota) comprises the most xerotolerant, xerophilic and also halophilic species worldwide. *Wallemia* spp. are found in various osmotically challenged environments, such as dry, salted, or highly sugared foods, dry feed, hypersaline waters of solar salterns, salt crystals, indoor and outdoor air, and agriculture aerosols. Recently, eight species were recognized for the genus *Wallemia*, among which four are commonly associated with foods: *W. sebi*, *W. mellicola*, *W. muriae* and *W. ichthyophaga*. To date, only strains of *W. sebi*, *W. mellicola* and *W. muriae* have been reported to be related to human health problems, as either allergological conditions (e.g., farmer’s lung disease) or rare subcutaneous/cutaneous infections. Therefore, this allergological and infective potential, together with the toxins that the majority of *Wallemia* spp. produce even under saline conditions, defines these fungi as filamentous food-borne pathogenic fungi.

## 1. Introduction

Low availability of water is one of the most life-limiting factors, and only specially adapted organisms can cope with such stress in their environment. For this reason, reduced water through drying or solute addition (e.g., salt, sugar) has been used for millennia for the conservation of food. Indeed, with water availability measured as water activity (a_w_) < 0.9, growth of the majority of bacterial pathogens is prevented [1]. However, many fungi can still thrive at much lower a_w_ (~0.8), and some even define the lowest a_w_ that has been seen to support life (0.61) [2,3]. These fungi are in general referred to as xerophilic, or more accurately, osmophilic, if they can grow in solutions with high solute concentrations, such as high sugars or salts. Those that grow under high salt are also known as halotolerant and halophilic if the main solute is NaCl, or chaotolerant and chaophilic if a chaotropic salt such as MgCl_2_ prevails [4]. Although xerophily and halophily are rare in the phylum Basidiomycota, the *Wallemia* spp. represent one of the most xerophilic fungal taxa, and include the most xerophilic, osmophilic, and even halophilic and chaophilic microorganisms described to date [5,6,7]. The ecology of these fungi is in line with their xerophilic character, whereby the majority of habitats from which strains of *Wallemia* spp. have been isolated are osmotically challenging, as either extremely dry and saline environments, and/or highly salted or sugared foods [5,6,7,8,9].

The extremophilic nature of *Wallemia* spp. is also illustrated by the initial description of these fungi when they were found along the Atlantic coast of Norway, as reported back in the year 1887 [10]. In the 18th and 19th centuries, the majority of the fish caught along this coast was cod (*Gadus* spp.), sold as klipfish. This was preserved with plenty of salt, and left to dry on the rocks beneath the cliffs, to also provide food for consumption outside the hunting season [10]. However, this conservation method did not prevent the growth of all microorganisms as regular spoilage occurred. A fish inspector named Mr. Wallem first tackled this problem at the end of the 19th century by taking samples of the contaminated fish to established Scandinavian scientists of the time, to try to discover what caused this spoilage [11]. The mycologist Johan Olav Olsen described the fungal contaminant as *Wallemia ichthyophaga* (Johan-Olsen, 1887), which was thus named after the fish inspector and indicated its ecological niche as “*ichthyophaga*”, literally meaning “eating fish” [10]. Unfortunately, none of the original material was preserved, and the neotype is now based on the strain isolated from the extremely saline waters of the Sečovlje solar salterns (Slovenia). Recently, strains of *W. ichythophaga* were again isolated from this original source of klipfish, with obvious fungal growth collected in Nordland, Lofoten (Norway) [12].

Strains of *Wallemia* spp. were later isolated not only from salted cod, but also from all sorts of sweet, salted and dried foods, as well as from the atmospheric air and from the hypersaline waters of solar salterns [5]. Indeed, *Wallemia* spp. are now considered to be important spoilage organisms for foods with low a_w_ [3,9]. However, these fungi of the genus *Wallemia* might not only result in economic losses due to food spoilage, as they might also represent a largely overlooked health risk. This is due to their production of mycotoxins that have both known and yet-to-be defined effects on human and animal health if they are consumed as part of contaminated foods and feed. These mycotoxins can cause the allergological problem known as “farmer’s lung disease”, and can also promote infections in immunocompetent humans [13].

This review focuses on the latest contributions to the phylogenetic resolution, ecophysiology, and pathogenic and mycotoxigenic potential of strains of the genus *Wallemia*.

## 2. The Phylogenetic Enigma of the Genus *Wallemia*

The long-lasting phylogenetic and taxonomic obscurity of the genus *Wallemia* was resolved in several studies that combined the old taxonomic literature and modern concepts based on molecular data [5,14,15]. The first comprehensive study here resolved the new class of Wallemiomycetes and order of Wallemiales into the phylum Basidiomycota, based on their unique type of conidiogenesis and their extreme xerotolerance [5]. Their phylogenetic position was supported by additional molecular analyses that described the class Wallemiomycetes as an early diverging basidiomycetous lineage with a basal position near the Entorrhizomycetidae, and possibly as a sister group to the Agaricomycotina and Ustilaginomycotina [14]. They are closely related to Agaricomycotina [16], as additionally confirmed in recent phylogenomic studies based on a 71-protein dataset [17] and on the whole proteomes [18]. These analyses support Ustilaginomycotina as a sister group to the branch that unites Wallemiomycetes and the remaining Agaricomycotina, and Pucciniomycotina as a sister group to the rest of the Basidiomycota [17,18]. Most recently, the evolutionary history of the genus *Wallemia* was resolved by its allocation to the newly distinguished sub-phylum Wallemiomycotina that split away approximately 487 million years ago [15]. In this way, the distinctiveness and uniqueness of the genus *Wallemia* was emphasised almost to the highest taxonomic rank.

Long after the initial definition of the single species of *W. ichthyophaga* in the genus *Wallemia* [19], two additional species were described: *W. sebi* and *W. muriae*. These were based on the differences in sequence data of the internally transcribed spacer region of their rDNA, and on their conidial size and xerotolerance [5]. Later on, a multi-locus phylogenetic approach resolved four new species [8]: *W. mellicola*, *W. canadensis, W. tropicalis* (closely related to *W. sebi*) and *W. hederae* (phylogenetic sister of *W. ichthyophaga*). Recently, another new species was added to the genus: *W. peruviensis*, which is closely related to *W. hederae* and was isolated from an agricultural setting in Peru [20].

The *Wallemia* spp. differ in size of conidia, xerotolerance, halotolerance (i.e., tolerance to NaCl), chaotolerance (i.e., tolerance to MgCl_2_), growth temperature range, extracellular enzyme profile, and secondary metabolite production [6,8].

The dichotomous keys on the basis of macromorphological, micromorphological and physiological characteristics of the species of the genus *Wallemia* recognised to date are available in Jančič et al. (2015) [8] and Diaz-Valderrama et al. (2017) [20].

## 3. The Most Xerophilic Fungal Genus Known to Date

*Wallemia sebi* is the most-studied species among the *Wallemia*, and is one of the most xerophilic fungi found worldwide, which is due to its dissemination through the atmospheric air and its colonization of diverse substrates with low a_w,_ and it is common on various types of foods (see Table 1). Its optimal a_w_ for growth is 0.97–0.92, and it has an extremely halotolerant and chaotolerant character, with optimal growth at 4% to 12% (*w*/*v*) NaCl, and 4% to 6% MgCl_2_. *W. sebi* can, however, also grow at up to 28% NaCl and 17% MgCl_2_ [8].

Compared to *W. sebi*, the phylogenetically close relative *W. mellicola* differs in terms of its larger size of conidia and lower halotolerance and chaotolerance. Growth of *W. mellicola* is still supported to the maximal concentrations of 24% NaCl and 13% MgCl_2_. Also, it has a worldwide distribution and can be isolated from different habitats, including soil, air and house dust, hypersaline water of solar salterns, salted, sugared and dried food products, seeds, straw, pollen and forest plants (see Table 1 for details).

Unlike the above (xerotolerant) species, *W. muriae* and *W. ichthyophaga* have the obligatory demand for lowered a_w_ in their habitats as they only grow on media supplemented with additional solutes like salts and sugars. The growth range of *W. ichthyophaga* is 9% to 30% NaCl [7], whereas *W. muriae* thrives in media supplemented with 4% to 25% NaCl [21] and tolerates up to 14% MgCl_2_ [6]. *W. ichthyophaga* is the most halophilic fungus described to date, with a growth optimum between 15% and 20% NaCl [7,22]. Furthermore, strains of *W. ichthyophaga* can also tolerate the highest concentrations of MgCl_2_ that still support active life (up to 20% MgCl_2_) [4].

Also the phylogenetic sister of *W. ichthyophaga*, *W. hederae* is clearly halophilic, with a growth optimum of 12% to 20% NaCl. However, it has no obligatory demand for lowered a_w_, as it can also grow, albeit poorly, on media without extra solutes [6]. *W. hederae* is distinctive from its relatives by large amounts of exudate formed in cultures that are composed of enzymes and secondary metabolites [6]. The ecological occurrence of *W. ichthyophaga* is in agreement with its halophilic behaviour, as it is strictly limited to extremely saline habitats like the hypersaline waters of salterns, salt crystals and salted meat and fish. In contrast, to date, *W. hederae* has only been isolated from non-saline substrates, like oak honey, barley seeds, hay and ivy pollen [6]. *W. muriae* is a common food contaminant of sugared and salted food products, and is found in the natural habitats of hypersaline waters of salterns world-wide [5].

*Wallemia canadensis* is clearly distinguished from *W. sebi* by its lower temperature range for growth (optimal growth temperature, 24 °C; compared to other related species as 30 °C), and lower halotolerance (NaCl, 0–24%) and chaotolerance (MgCl_2_, 0–11%). The ecology of *W. canadensis* appears to be limited to temperate and cold environments, where it has been isolated from house dust and soil. On the contrary, the other species in close phylogenetic proximity to *W. sebi*, *W. tropicalis*, appears to be limited to soils and air in subtropical climates [8]. Finally, *W. peruviensis* was originally defined from a restricted geographical location, as from air of an agricultural setting in Peru, and it is closely related to *W. hederae*. It is also halotolerant, with growth up to 17% NaCl, and chaotolerant, as it can tolerate up to 16% MgCl_2_; *W. peruviensis* produces less exudates compared to *W. hederae* [20].

Among the eight *Wallemia* spp., *W. sebi*, *W. mellicola*, *W. muriae* and *W. ichthyophaga*, have been commonly associated with food habitats, while the rarely encountered *W. hederae* has been linked to oak honey. There are only few recent (after the year 2014) publications on *Wallemia* spp. associated with food: a case report on contamination of maple syrup [23] and wheat grains [24], being a dominant fungus on stored green coffee beans [25] and found also on edible insects (crickets and locusts) [26]. Other recent publications [6,8,9,23] mostly reclassify previously isolated *Wallemia sebi* from various habitats including foods into newly described species, for instance, as *W. mellicola* and *W. muriae* (see Table 1).

## 4. Potential Health Risks of the Bioactive Metabolites of *Wallemia* spp.

Secondary metabolites [29,30,31] do not only have crucial roles in interactions with other organisms, as they can also be involved in nutrition, sporulation and even stress tolerance [32]. The diversity of the bioactive metabolites identified in the extremotolerant/extremophilic fungi studied to date is relatively low. Interestingly, an in-silico analysis of the genomic data of *W. mellicola* (previously known as *W. sebi* [33]) and *W. ichthyophaga* revealed only a few secondary metabolite clusters, whereas wet laboratory data have indicated a considerable number of secondary metabolites that can be synthesized under different conditions [27].

The knowledge of the mycotoxigenic repertoire of *Wallemia* spp. was until recently limited only to *W. sebi*. *W. sebi* was reported to produce a number of bioactive metabolites/toxins, which include the tricyclic dihydroxysesquiterpenes wallimidione, walleminone and walleminol (walleminol A), and the azasteroids UCA 1064-A and 1064-B (reviewed in [27,34]). Among these, walleminol is the only one of these metabolites to be detected in food to date, in samples of jam and cake shown to be contaminated with *W. sebi*. Walleminol is toxic to brine shrimp and the protozoa *Tetrahymena pyriformis*. No toxic effects are known for UCA 1064-A and 1064-B. In contrast, they have shown antitumor, antiproliferative and antimicrobial activity [35]. There are only a few reports on secondary metabolite production under saline growth conditions [35,36,37,38].

A recent comprehensive investigation was carried out into the secondary metabolites produced by seven of the species of the genus *Wallemia* (i.e., *W. sebi*, *W. muriae*, *W. mellicola*, *W. tropicali*, *W. canadensis*, *W. hederae*, *W. ichtyhophaga*) under osmotically unstressed and stressed conditions [27]. This identified a mixture of toxic metabolites that included walleminol, walleminone and wallimidione, and contrary to common belief, their production was increased in response to increased concentrations of NaCl [27]. This behaviour makes *Wallemia* spp. an important mycotoxigenic contaminant of salt-preserved foods, and this bioactive potential of *Wallemia* spp. might thus have serious undesirable effects on human health. Further studies on the toxicities of these bioactive metabolites in vertebrates are needed to better define these effects, and at present, the contamination of salted food with *Wallemia* spp. should be considered with caution in terms of food quality control [27].

## 5. Infectious and Allergological Cases Linked to *Wallemia* spp*.*

It is now becoming clearer that extremophilic fungi can present a threat to human health through their stress-tolerance mechanisms that appear designed to promote their pathogenicity. Indeed, the link between extremophilic lifestyle and pathogenicity is seen in several species of pathogenic fungi that have extremotolerant/extremophilic close relatives [29].

Little information is available on the pathogenicity of *Wallemia* spp. and the associated clinical features, potentially because these infections rarely occur, or because they have remained under-diagnosed. Indeed, *Wallemia* spp. are slow growing, and they require media supplemented with high amounts of salts to promote their competitive advantage against other fungi. Therefore, *Wallemia* isolates can be easily overgrown by other moderate xerophilic fungi, or simply overlooked because of short cultivation times. This also appears to be the reason why there are relatively few *Wallemia* strains deposited in culture collections, albeit they show a ubiquitous and world-wide distribution.

Evidence of the involvement of *Wallemia* spp. in human infections and pathogenesis has so far been limited to only *W. sebi*, which was until 2005 the only known species of this genus [5]. Only three reports of cutaneous and subcutaneous infections have been linked to *Wallemia* spp. The first two of these did not indicate any clinical features, and were reported in 1909 and 1950 as ‘hemisporiosis’, after the synonymous species *Hemispora stellata*. The third occurrence was reported in 2008, as a case of subcutaneous phaeohyphomycosis in a 43-year-old woman from northern India who was otherwise healthy and immunocompetent. She suffered from a non-healing ulcer on the dorsum of a foot that started as an itchy papule, although with no prior injury. Over 8 months this gradually developed into an erythematous lesion. Diagnosis and pathogen identification was based on histological demonstration of septate hyphae and recovery of the fungus in cultures [13]. Subsequent phylogenetic analysis reclassified this pathogenic *W. sebi* strain to the species *W. mellicola* [8]. However, it is important to note that it is only rarely that pathogenic fungi cause infections in immunocompetent individuals. Thus, this this case report should be considered as a warning sign of the pathogenic potential of *Wallemia* spp. However, there are no recent (after the year 2014) clinical reports linked to *Wallemia* spp. as the causative agent of infection.

However, *Wallemia* spp. are important human allergens, as they can produce masses of tiny propagules that are ideal for aerial dispersal, and can thus enter into the lungs of hosts. Cell size is an important factor for effective invasion and for establishing an infection in the pulmonary system [29]. The conidia of *Wallemia* spp. are very small, with a range from 1.5 µm to 3.0 µm [8], and they thus fulfill this criterion. Another important character that allows the spores to escape inflammatory immune responses and to successfully colonize the lungs is the presence of layers of hydrophobins [39], which are small cell-wall proteins with amphipathic properties that cover the antigens of the fungal cells [40]. Indeed, genome analysis of *W. mellicola* (previously designated as *W. sebi*) and *W. ichythophaga* uncovered an enrichment of genes that encoded these hydrophobins [18], which might contribute to their pathogenic and allergenic potentials.

Another important view of the involvement of fungi in the regulation of immune responses was recently highlighted [41]. Evidence has been provided that the “healthy” fungal community in the gut has an important role in the function of the immune system, and that prolonged antifungal treatments can increase the severity of colitis, and can also worsen the development of allergic airway disease, mainly because of the changes to the fungal community. *Wallemia* spp. was reported also as a member of fungal microbiota in human gut [42]. Prolonged antifungal therapies have been shown to reduce the levels of *Candida* spp. and to increase the proportions of fungal species that are otherwise absent or only present at low levels in healthy untreated mice, such as *Wallemia* spp. (together with *Aspergillus* *spp.* and *Epicoccum *spp.).** When these mice were fed with a mixture of three such fungi, as *A. amstelodami*, *E. nigrum* and *W. sebi,* the effects of the exacerbated features of allergic airway disease were seen to be similar to those seen after antifungal treatment [41,42].

Chronic inhalation of spores and conidia from mouldy hay, straw or grain might therefore cause the bronchial asthma [43,44] or hypersensitivity pneumonitis (i.e., extrinsic allergic alveolitis) that is known as farmer’s lung disease, and that can be combined with pulmonary fibrosis and emphysema [45]. The symptoms can include dyspnoea, cough, tiredness, headaches and occasional fever/night sweats, and these depend on the severity of the farmer’s lung disease [46,47,48,49,50]. Numerous studies have revealed high concentrations of *Wallemia* spp. in air and dust in outdoor and indoor environments, as well as in the air of agricultural environments [6,20,51,52]. Indeed, for the air in stables and hay barns in Slovenia and Denmark, propagules of *Wallemia* spp. reached the highest recorded levels of 500 to 10^6^ colony forming units (CFU)/m^3^, compared to the 20 to 500 CFU/m^3^ reported for residential buildings [6]. *W. sebi* has frequently been reported to be the cause of this respiratory allergy and of atopic diseases of asthmatic individuals [53,54]. However, the dominant species among *Wallemia* spp. in human environments in central Europe has been reported to be *W. muriae* [6], and not *W. sebi*, as was previously reported. For this reason, *W. muriae* can be considered as the primary causative agent of farmer’s lung disease and other allergological problems [6].

## 6. Final Remarks

To date, only three species from the genus *Wallemia* have been involved in human health issues: *W. sebi*, *W. mellicola* and *W. muriae*. These include allergological diseases such as farmer’s lung disease, and even, albeit rarely, some subcutaneous and cutaneous infections. The case of the subcutaneous infection due to *W. mellicola* (initially determined as *W. sebi*) is especially intriguing, because it occurred on an immunocompetent patient with no prior injury around the phaeohyphycosis that developed. Recently, it was also suggested that due to the high aerial abundance of *W. muriae*, this should be considered as the causative agent of farmer’s lung disease. The indications are thus that infections with *Wallemia* spp. can have serious effects on human health, not only via inhalation, but also via the consumption of contaminated food. It has already been shown in mice that over-representation of *Wallemia* spp. in the gut contributes to increased severity of asthmatic bronchial diseases and colitis. In addition, many *Wallemia* spp. can produce a rich repertoire of secondary metabolites that includes mycotoxins, especially when they thrive under high saline conditions. All of these are important aspects related to potential health risks that need to be considered when assessing the quality and safety of food preserved with salt and contaminated with *Wallemia* spp.

## Figures and Tables

**Table 1:** Known habitats, geographic distributions, and pathogenic potentials of the eight members of the genus *Wallemia*. Adapted after [5,6,8,9,13,23,24,25,26,27,28].

*Wallemia* spp.	Habitat	Geographic Distribution	Pathogenic Incidence
*W. sebi*	Hypersaline water in solar salterns and salt lakes; hay; sea salt; air and dust in indoor environments (house, office, storage areas); pond water, mineral water; seeds (sunflower, wheat, rye, barley, maize, in-shell peanuts, pecans, peas); baked goods (bread, ginger bread, marzipan cake); beans (mung, soybeans and soy products, green coffee beans); cereals (corn, rice, wheat); chocolate, milk and condensed milk; chili and peppers, fruits and fruit products (dates, jams, jellies, dried prunes, sultanas); maple syrup, dried salted fish, meat products, suet	Worldwide (Africa, Asia, Europe, North America)	Chronic ulcerative skin lesion in man (one case reported, Groningen, The Netherlands); fatal livestock toxicosis associated with contaminated hay (one case reported, Berkshire, UK)
*W. mellicola*	Salty foods (peanuts, dried fish); sugared food (date honey, cakes, jam, maple syrup, chocolate); dried food (bread, coconut pulp); hypersaline waters of solar salterns; air, dust and surfaces in indoor environments; soil; forest plants; seeds, straw, pollen	Worldwide (Asia, Europe, North America, Middle America, South America, Micronesia)	Subcutaneous lesion (phaeohyphomycosis) on foot in an immunocompetent human patient (Varanasi, Uttar Pradesh, India)
*W. muriae*	Sugared food (date honey, cake, chocolate); salty food (peanuts); edible crickets and locusts; hypersaline waters of salterns world-wide; dry substrates (straw, seeds); air in agricultural and human associated environments; an insect (one report)	Worldwide (Asia, Europe, North America, South America)	Farmer’s lung disease; bronchial asthma
*W. ichthyophaga*	Hypersaline waters of salterns in Slovenia and Namibia; salted meat and fish; klipfish (salted cod)—not recorded from environmental substrates with high sugar content	Sporadic (Slovenia, Norway, Namibia)	No reports
*W. tropicalis*	Soil; house dust	Subtropical and tropical climates (Egypt, Uruguay, Indonesia and Micronesia)	No reports
*W. canadensis*	Cedar swamp; catwalk in silos; indoor dust and air	Temperate and cold climates (Canada, UK, Finland)	No reports
*W. hederae*	Common on ivy flowers (pollen); oak honey, barley seeds, hay, green coffee beans	Southern Europe (Slovenia, Croatia), South America (Mexico)	No reports
*W. peruviensis*	Air in agricultural settings	South America, Peru	No reports
